# The positional demands of explosive actions in elite soccer: Comparison of English Premier League and French Ligue 1

**DOI:** 10.5114/biolsport.2025.139083

**Published:** 2024-05-24

**Authors:** Ryland Morgans, Wonwoo Ju, John Radnor, Piotr Zmijewski, Ben Ryan, Chris Haslam, Matthew King, Ronan Kavanagh, Rafael Oliveira

**Affiliations:** 1 School of Sport and Health Sciences, Cardiff Metropolitan University, Cardiff, UK; 2 High Performance Group, Korea Football Association, Republic of Korea; 3 Jozef Pilsudski University of Physical Education in Warsaw, 00-809 Warsaw, Poland; 4 Research and Development Center Legia Lab, Legia Warszawa, Poland; 5 Brentford FC Football Research Centre, Brentford FC, London, UK; 6 Football Performance Hub, Institute of Coaching and Performance, University of Central Lancashire, Preston, UK; 7 Research Centre in Sports Sciences, Health and Human Development, 5001–801 Vila Real, Portugal; 8 Sports Science School of Rio Maior – Instituto Politécnico de Santarém, 2040–413 Rio Maior, Santarém District, Portugal

**Keywords:** Football, Accelerations, Decelerations, English Premier League, French Ligue 1, High-intensity actions

## Abstract

The aims of the present study were to: (i) quantify accelerations and decelerations of soccer players during match-play across two consecutive seasons from the English Premier League (EPL) and Ligue 1 (L1); and (ii) compare any positional differences between the two leagues. Fifty-eight male professional soccer players were monitored during all league matches (n = 144) across seasons 2020/21 and 2021/22. The absolute number of accelerations (> +3 m/s^−2^) and decelerations (< -3 m/s^−2^) and accelerations and decelerations per minute were examined. The relative number of accelerations and decelerations across all positions was higher with moderate effect sizes in the EPL when compared to L1 (p < 0.001, for both). Significant differences were observed in accelerations and decelerations across all playing positions (p < 0.001 and p = 0.001 respectively, with moderate to very large effect sizes), except for centre forwards (CF) (accelerations p = 0.40; ES = 0.16; decelerations p = 0.97; ES = 0.01). This study provides valuable insights into the positional acceleration and deceleration differences in the EPL and L1, which should be considered in match running performance evaluations. While confirming higher accelerations and decelerations in the EPL, the unique case of CF challenges current evidence, emphasising the need for a more granular understanding of the positional demands of explosive actions incorporating accelerations and decelerations in elite soccer.

## INTRODUCTION

Soccer is a sport that demonstrates its intermittent nature through short bursts of high-intensity activity interspersed with longer periods of low-intensity actions [1]. Benchmarking and profiling these physical characteristics is important to physically prepare players for the demands of match-play through effective training programmes [2]. Due to the adaptation of modern technology in soccer [3], the use of tracking-based technologies such as the Global Positioning System (GPS) has grown significantly. This has improved the ability of applied practitioners to profile the physical demands of players from competitive matches and training, which has facilitated more precise training prescriptions, load modifications, and thus, better preparation of players for match-play [4–6].

There has been a noticeable increase in high-intensity activities during competition worldwide over the last few decades [7–9]. Early work suggested that sprint distance and the number of sprints increased by ~35 and ~85% respectively, while mean sprint distance was lower in 2012/13 compared to 2006/07 with the proportion of explosive sprints increasing [7]. More recently, the trend of increased running demands has been observed following the 2022 World Cup tournament [10]. Distances covered at higher intensities were 16–92% and 36–138% higher for wide midfielders and wide forwards compared to central defenders, defensive and central midfielders, as well as centre forwards [10]. While defensive and central midfielders covered a greater proportion of distance at higher intensities out-of-possession (71–83%), and attacking mid-fielders, wide, and centre forwards covered more in-possession (55–68%) [10]. Moreover, high-intensity actions (e.g., sprinting, accelerating, and decelerating) significantly influence decisive moments of the match [11, 12]. Consequently, high-intensity movements in match-play have gained more attention [13].

Even so, researchers have a tendency to examine total, high-speed running and sprint distance in isolation, without considering acceleration and deceleration movements [14]. These high-intensity actions induce not only physiological but also mechanical demands, accounting for ~10% of total workload of elite soccer players during match-play, irrespective of playing position [15]. Additionally, the number of accelerations during match-play is up to ~8 times higher than sprint actions (~90–120 vs ~15–30, respectively) [16, 17], while deceleration actions occur as often as acceleration actions, leading to an even greater mechanical load [15]. Thus, it appears crucial for practitioners to profile such high-intensity actions throughout a season so that effective preparation and recovery can be implemented to allow players to cope with the physiological and mechanical demands of match-play.

Given the fact that physical characteristics of players vary across playing positions [18, 19] as well as different leagues [20], it would be insightful to compare differences between playing positions and various elite soccer leagues. In this regard, previous research examining data from the season 2006/07 showed that English Premier League (EPL) players displayed higher high-intensity running distance in matches than La Liga players irrespective of playing position [20]. More recently, another study [21] compared Portuguese and Dutch second leagues and found that Portuguese players produced higher total and sprinting training distances, although no comparisons during match-play or among playing positions were considered. Numerous publications [6, 9, 19] are available covering reference ranges for basic running characteristics of soccer players and various contextual variables. Nonetheless, no previous studies considered accelerations and decelerations [20, 21], thus reinforcing the relevancy of the present study as it analysed two different countries/leagues and positional differences which is vital to improve coaches’ knowledge of various training methods to enhance player preparation and recovery [22]. Where these data are utilised in the scouting assessment of soccer players, by coaches to design training, and by performance staff and physiotherapists to develop individual recovery protocols. Therefore, the aims of the present study were to: (i) quantify accelerations and decelerations of soccer players during match-play across two consecutive seasons from the EPL and French Ligue 1 (L1); and (ii) compare any positional differences between the two elite European soccer leagues. Based on previous literature [20], the study hypothesis was that the EPL team would present a higher number of accelerations and decelerations during match-play.

## MATERIALS AND METHODS

### Design

This longitudinal study over two consecutive seasons (2020/21 and 2021/22) involved professional soccer players from two European teams, the EPL and L1. Match acceleration and deceleration performance variables were collected using a GPS (Apex Pod, Statsports; Northern Ireland, UK). Data from all competitive matches across both leagues during two seasons were analysed. A non-probabilistic sampling protocol was employed to recruit participants. During the observation period, consistent player monitoring approaches were implemented without any interference from the researchers.

### Participants

Data from both seasons included 58 male players (EPL: age 23.2 ± 5.9 years, weight 75.2 ± 8.1 kg, height 1.83 ± 0.06 m; L1: age 24.3 ± 5.1 years, weight 76.6 ± 8.5 kg, height 1.83 ± 0.07 m). The data was obtained from all official matches played during both seasons (EPL n = 38, L1 n = 34). The EPL team adopted a 4-3-3 or 3-5-2 formation and implemented a hybrid model of possession that included possession-based and direct-play strategies. While the L1 team consistently implemented a 4-3-3 formation and also adopted a mixed approach of tactical strategies when in possession. Furthermore, for both study teams when out of possession a mixture of high-press and mid-block (a narrow and compact team shape defending the middle third of the pitch) strategies were employed. The research inclusion criteria have been previously applied [22] and were: (i) named in the first-team squad at the start of both study seasons, (ii) played in at least 80% of matches, and (iii) only completed official team training during the study period. Additionally, the exclusion criteria for the study have also been previously employed [22] and included: (i) long-term (three months or longer) injured player data, (ii) joining the team late in either of the study seasons, (iii) lack of full, complete match data, (iv) an in-sufficient number of satellite connection signals, and (v) goalkeepers, due to the specific nature of match activity and low running demands [23].

Only outfield players who completed the entire match (≥ 90 min) were included for analysis. Players were assigned to one of five playing positions as match demands for these differ significantly. The methodology of differentiating specialised positions was adapted from previous research [24]. Participants were classified as: EPL full-backs (FB; n = 3), centre back (CB; n = 5), centre midfielders (CM; n = 7), attacking midfielders (AM; n = 4), and centre forwards (CF; n = 4); L1 full-backs (FB; n = 7), centre back (CB; n = 6), centre midfielders (CM; n = 10), attacking midfielders (AM; n = 5), and centre forwards (CF; n = 7). The small sample size is supported by previous studies in soccer [22, 23]. Even so, the power of the sample size was calculated through G-Power [25]. Post-hoc analysis was conducted considering the study aims. For comparison analysis, an F-test, with a total of 58 participants with a p = 0.05 and effect size of 0.1 was performed. The actual power achieved was 86%.

All data collected resulted from normal analytical procedures regarding player monitoring over the competitive season, nevertheless, written informed consent was obtained from all participants. All data were anonymised prior to analysis in accordance with the Declaration of Helsinki. Moreover, this study was approved by the local ethics committee of the University of Central Lancashire and the professional clubs from which the participants volunteered [26].

### Data Collection Procedures

Data were collected from all (n = 144) in-season matches played by the examined teams across the two study seasons. The examined EPL and L1 team participated in a total of 76 and 68 matches respectively across the study seasons.

Accelerometer match data were consistently monitored across the study seasons using an 18 Hz GPS technology tracking system (Apex Pod, version 4.03, 50 gr, 88 × 33 mm; Statsports; Northern Ireland, UK) that has previously provided good to moderate reliability (coefficient of variation (CV) = 0.1 to 3.9%) for the majority of thresh-old-based accelerations and decelerations [27]. The 18 Hz system has also shown good validity and reliability for determining the distances covered (typical error of estimate (TEE): 1.6–8.0%; CV: 1.1–5.1%) and sprint mechanical properties (TEE: 4.5–14.3%; CV: 3.1–7.5%) [28]. All data collection procedures and unit error and reliability have previously been reported [29, 30]. Following every match, accelerometry data were extracted using proprietary software (Apex, version 4.3.8, Statsports Software; Northern Ireland, UK) and exported to a secure database for analysis, as software-derived data is a more simple and efficient way for practitioners to obtain data in an applied environment, with no differences reported between processing methods (software-derived to raw processed) [30].

Variables analysed were selected based on previous publications [22, 31, 32] and were analysed as absolute (total number) and relative data (divided by actual playing time for each player). Thus, the total number of accelerations and decelerations and the number of accelerations (> +3 m/s^−2^ with minimum duration of 0.5 s) and decelerations (< -3 m/s^−2^ with minimum duration of 0.5 s) per minute [33, 34] were examined.

### Statistical Analysis

Descriptive data (mean ± SD) were determined for the number of accelerations and decelerations per minute for the different positions (CB, FB, CM, AM, and CF) and leagues (EPL and L1). Homogeneity of variance was assessed via Levene’s statistic and, where violated, Welch’s adjustment was used to correct the F-ratio. Two-way (5 × 2) analysis of variance (ANOVA’s) were conducted to identify differences in the number of accelerations and decelerations per minute across different positions and leagues. Post-hoc analysis was used to identify the positions that were significantly different to one another using either Bonferroni or Games-Howell post-hoc analyses, where equal variances were and were not assumed, respectively.

Effect size (*η*^2^) values were reported for the ANOVA results, while Cohen’s *d* values (*d*) were reported for significant post-hoc results. *η*^2^ values in the range 0–0.009 are considered insignificant effect sizes, 0.01–0.0588 as small effect sizes, 0.0589–0.1379 as medium effect sizes, and values greater than 0.1379 as large effect sizes. Cohens *d* effect size magnitudes were interpreted using the following classifications: trivial < 0.19; small 0.2–0.59; 0.6–1.19 moderate; 1.2–1.9 large; 2.0–3.9 very large; > 4.0 extremely large [35]. All significance values were accepted at p < 0.05 and all statistical procedures were conducted using JASP (version 0.18) for Macintosh.

## RESULTS

**FIG. 1:**
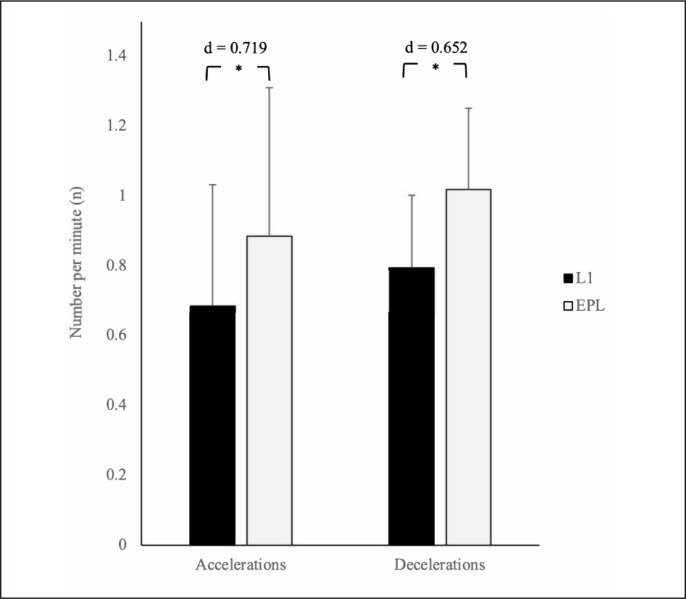
Difference in number of accelerations and decelerations per minute between the EPL and L1. * indicates significant difference between leagues (p < 0.001)

Descriptive statistics for the number of accelerations and decelerations for the whole team are presented in Figure 1. The two-way ANOVA identified a significant main effect for position (p < 0.001; *η*^2^ = 0.034) for the number of accelerations per minute. Full-backs and AM completed more accelerations per minute than CB and CM (p < 0.001–0.019; *d* = 0.319–0.499), while CF also completed more than CB (p = 0.003; *d* = 0.440). For the number of decelerations per minute, there was also a significant main effect for position (p < 0.001; *η*^2^ = 0.076), where AM, CM, and FB completed more decelerations per minute than CB (p < 0.001; *d* = 0.621–0.847). Full-backs and CM also completed more decelerations per minute than CF (p = 0.001–0.032; *d* = 0.350–0.513).

There was also a significant main effect for league for number of accelerations per minute (p < 0.001; *η*^2^ = 0.094) and number of decelerations per minute (p < 0.001; *η*^2^ = 0.075). Players from the EPL performed significantly more accelerations and decelerations per minute than L1 players (*d* = 0.719 and 0.652, respectively).

Descriptive statistics for the number of accelerations and decelerations per minute for each position across both leagues are presented in Table 1. There was no significant interaction effect between league and position for the number of accelerations per minute (p = 0.901; *η*^2^ = 0.001), or the number of decelerations per minute (p = 0.104; *η*^2^ = 0.007). However, when considering the number of accelerations per minute, AM from the EPL completed more than CB in the EPL (p = 0.028; *d* = 0.535), and all positions in L1 (p < 0.001; *d* = 0.726–1.250), while FB from the EPL also completed more accelerations per minute than all positions from L1 (p < 0.001; *d* = 0.724–1.228). Centre backs, CM, and CF from the EPL completed more accelerations per minute than CB and CM from L1 (p < 0.001; *d* = 0.569–1.071). Finally, CF and FB from L1 completed more accelerations per minute than CB from L1 (p < 0.001; *d* = 0.724 and 1.228, respectively).

**TABLE 1:** Comparison of the number of accelerations and decelerations per minute between the EPL and L1 for each position.

Position	League	Accelerations per minute (n/min)	Decelerations per minute (n/min)
CB	L1	0.60 ± 0.28	0.61 ± 0.28
	EPL	0.81 ± 0.15^c,d^	0.86 ± 0.15^c^

FB	L1	0.74 ± 0.37^c^	0.89 ± 0.49^c^
	EPL	0.96 ± 0.19^a^	1.17 ± 0.13^a,b^

CM	L1	0.64 ± 0.36	0.81 ± 0.47^c^
	EPL	0.87 ± 0.19^c,d^	1.14 ± 0.19^a,b^

AM	L1	0.73 ± 0.34	0.86 ± 0.40^c^
	EPL	0.97 ± 0.29^a,b^	1.04 ± 0.30^a,b^

CF	L1	0.75 ± 0.35^c^	0.79 ± 0.39^c^
	EPL	0.92 ± 0.12^c,d^	0.91 ± 0.12^c^

a = significantly different to all L1 positions (p < 0.05);

b = significantly different to EPL CB (p < 0.05);

c = significantly different to L1 CB (p < 0.05);

d = significantly different to L1 CM (p < 0.05).

Note: CB = centre back; FB = full back; CM = centre midfielder; AM = attacking midfielder; CF = centre forward

The number of decelerations per minute was greater for AM compared to CB in the EPL (p = 0.033; *d* = 0.527), and all positions in L1 (p < 0.001; *d* = 0.506–1.221). Centre midfielders and FB completed more decelerations per minute than CB from the EPL (p < 0.001; *d* = 0.798 and 0.893, respectively) and all positions from L1 (p < 0.001; *d* = 0.691–1.586). Finally, CF and CB from the EPL completed more decelerations per minute than CB from L1 (p < 0.001; *d* = 0.857 and 0.693, respectively).

## DISCUSSION

The aims of the present study were to: (i) quantify accelerations and decelerations of soccer players during match-play across two consecutive seasons from the EPL and L1; and (ii) compare any positional differences between the two elite European soccer leagues.

The main findings of the present study were that the relative total number of accelerations and decelerations were higher in the EPL when compared to L1 considering whole team data (p < 0.001 with moderate effect size for both variables). Since soccer involves the interaction of physical, technical and tactical actions among players, the adoption of differing technical/tactical strategies will result in distinct physical demands [36], which was confirmed by the present findings. This aligns with the perception and grounded research opinion that the EPL is characterised by a more physically demanding and fast-paced style of play, in particular adopting a more ‘direct’ style of play, with a higher efficient attack conducted in a short time duration [37]. This style may also be reinforced by a study examining the differences in fouls and cards administered as indicators of aggressive play in the Premier Leagues of England, France, Germany, Italy, and Spain and supported the notion that the EPL was the most aggressive league across Europe [38]. Nonetheless, no research was found considering the comparison of different leagues and playing positions based on accelerometry-based variables. Thus, more research is warranted to confirm such statements.

The analysis of positional acceleration and deceleration demands have recently been documented [39, 40], albeit not in elite European soccer players and not comparing league and positional differences. Similar to other research [17, 23] that reported CB perform the lowest number of accelerations during match-play, whilst WM execute the greatest compared to all other positions and the highest number of acceleration efforts was observed in FB [16]. The present study showed that CB performed less accelerations in both leagues. However, CF and FB performed more accelerations in L1 while FB and AM performed more accelerations in the EPL. Moreover, previous research showed that CB perform the lowest number of decelerations during match-play, whilst CF executed the greatest compared to all other positions [17]. The present study corroborated these previous findings [17] in terms of a low number of decelerations for CB, although in contrast to earlier work that found FB performed the highest number of decelerations [17]. It is relevant to mention that previous research only utilised data from La Liga which may cause some bias when interpreting results [17]. Even so, another study suggested that wide midfielders usually perform a high number of decelerations [41]. While the present study did include wide midfielders, FB were highlighted as players who covered a large area in the field being similar to midfielders, thus justifying the higher results.

The lack of identical results in both leagues challenges the prevailing notion of distinct physical demands [36]. Furthermore, it may also reflect the tactical strategy (offensively and defensively) of the examined teams that subject AM (in the EPL) and CF (in L1) to produce similar explosive actions when compared with FB during competitive match-play. Notwithstanding, when considering the total number of accelerations and decelerations, all positions showed higher values in the EPL team compared with the L1 team (although not all results were significant). Such findings reinforce the analysis of relative data of accelerations and decelerations per minute.

Additionally, although not examined in the present study, it is also possible that tactical formation, that contributes to explosive actions, may influence match result. For instance, a recent study compared the three best Spanish soccer teams examining running measures with and without ball possession considering different playing positions, where different formations and styles were employed: 4-4-2 formation with a compact defence and direct attack strategy; 4-3-3 formation with an indirect style of play; and 4-3-3 formation with intricate attacks and effective counterattacks [42]. While the authors found minimal differences between the three different formations, when considering the various positions, it was clear that team formation and the differing tactical demands have a significant influence on running performance [42]. Thus, it is speculated that accelerations and decelerations may also differ in this study as different playing position patterns were revealed. While contextualised high-intensity running profiles of elite soccer players with reference to general and specialised tactical roles have gained interest recently [18], there is no research specifically examining the quantity or quality of accelerations and decelerations across two different elite European leagues considering tactical development, thus further research is warranted.

Furthermore, if other contextual variables such as opposition standard, possession characteristics and match location were considered, there is the possibility that significant differences in relative accelerations and decelerations for CF of both leagues would be observed being the only position that significantly differed between leagues. For instance, it was recently shown that CF performed a significantly higher number of acceleration efforts against top-level teams when playing at home compared to away matches [40]. Additionally, the frequency of decelerations per minute was also position-specific [40]. In particular, the number of decelerations per minute performed by FB, CM, WM and CF were higher than CB, which is consistent with previous studies [16, 39], while CF were similar between the EPL and L1. These varying acceleration and deceleration results between studies may partly be explained by the effect of differing playing formations as positional differences are significantly affected by playing formations [32]. However, thoroughly examining the effect of tactical aspects such as in and out of possession strategies and team formation on explosive acceleration and deceleration actions seems problematic as the development of tactical nuances suggests that elite teams do not select and maintain the same strategies or formations throughout the whole match or season [40]. An approach that merges the tactical elements of match-play with the physical outputs warrants further investigation to allow a greater understanding of these demands that may be practically useful when designing position-specific drills and sessions to optimally prepare players.

### Practical Applications

The present results have some practical implications for coaches and performance staff in tailoring position-specific training regimes, load management and individualised recovery strategies based on differing leagues and positional requirements. The unique case of CF, when analysing relative data, suggests that, despite the overall differences in acceleration and deceleration actions between leagues, certain playing positions may share common physical demands irrespective of league context. However, this may be of greater interest when contextualising acceleration and deceleration behaviours with tactical variables as this may help practitioners design more effective training programmes [40]. Notably, it is relevant to highlight that this study utilised data from two seasons to compare two different leagues which seems to be the first research to consider this concept. Finally, it should be emphasised the importance of analysing relative per minute rather than absolute data.

### Limitations and Future Research

Several limitations should be noted when interpretating the findings of this study. The current data are reflective of the methods and practices of two elite soccer clubs from varying European leagues, however the positional match running performance and variations resulting from possession classifications and team formation [15, 43] were not considered for analysis. Also, this study did not examine the effects of standard opposition and match location. Consequently, the results should only be generalised to similar cohorts, level of competition, and tactical approaches as previously suggested [20]. Thus, future studies should be conducted to compare current findings utilising larger sample sizes with various team formations and possession time. Moreover, some studies reported that acceleration and deceleration metrics may have a high measurement error and variation [43]. Although, there are more recent research reporting such metrics [40, 44, 45]. In addition, this study did not consider the effects on running distances (e.g., total distance, HSR or sprinting) and effective playing time [46]. Such limitations suggest that future research should include these contextual factors as these variables can influence match outcome [18]. Furthermore, considering match outcome (win, draw, loss) can affect the quantity of accelerations and decelerations [44, 45] performed, this contextual variable should be included in future research. Also, further studies should focus on including acceleration and deceleration metrics into fatigue assessment and recovery protocols. Lastly, there were no analysis considering external factors such as time of day of matches and weather conditions (e.g., rain, wind, or temperature) which can also affect the findings of this study and should be considered in future studies when comparing different leagues and contexts.

## CONCLUSIONS

In conclusion, this study provides valuable insights into the nuanced differences in explosive actions across playing positions in the EPL and L1. While confirming the general trend of higher acceleration and deceleration frequencies in the EPL, the unique case of CF challenges current evidence, emphasising the need for a more granular understanding of positional demands of explosive actions incorporating accelerations and decelerations in elite soccer. Further research exploring the contextual and tactical factors influencing these patterns will contribute to a more comprehensive picture of the physical demands in elite European soccer.
